# Comparative study of dyslipidemia among pre and post - menopausal women

**DOI:** 10.6026/973206300211385

**Published:** 2025-06-30

**Authors:** Padmavathi P., Chirag K. Pandya, Samiya Begum, Gnanadesigan Ekambaram, Mahalakshmi B., Sivasubramanian N., Vijayabanu S.

**Affiliations:** 1 Department of Biochemistry, Nootan Medical College & Research Centre, Sankalchand Patel University, Visnagar-384315, Gujarat, India; 2 Department of Biochemistry, ACS Medical College and Hospital, Dr. M.G.R Educational and Research Institute, Maduravoyal, Chennai, India; 3 Department of Physiology, Nootan Medical College & Research Centre, Sankalchand Patel University, Visnagar-384315, Gujarat, India; 4 Department of Paediatric Nursing, Nootan College of Nursing, Sankalchand Patel University, Visnagar-384315, Gujarat, India; 5 Department of Psychiatric Nursing, Nootan College of Nursing, Sankalchand Patel University, Visnagar-384315, Gujarat, India; 6 Department of Medical & Surgical Nursing, Cheran college of Nursing, Coimbatore, Tamil Nadu DR.M.G.R Medical University, Chennai, India

**Keywords:** Dyslipidaemia, premenopausal women, post-menopausal women

## Abstract

Dyslipidaemia is an independent risk factor for cardiovascular disease in pre and post-menopausal women. Therefore, it is of interest to analyse the lipid abnormality among them. Totally n=214 women were recruited, among them (n=107) were premenopausal and (n=107) were postmenopausal women. The prevalence of dyslipidaemia in our study was 81.77% (n=175). Among premenopausal women 26.1% (n=28) and post menopause 35.5% (n=38) had isolated lipid abnormality whereas mixed dyslipidaemia was 37.3% (n=40) in premenopausal women and 64.4% (n=69) in postmenopausal women. Study shows the complexity of lipid profile variations and suggests the need for age and stage-specific cardiovascular risk assessment.

## Background:

Women, faces different stages in her reproductive system as an aging process in her life. Significant hormonal changes during menopause, linked to changes in metabolic profile [1]. As the woman ages, the risk of developing dyslipidaemia rises, the incidence of dyslipidaemia rises throughout a woman's lifespan with negative alterations occurring around menopause. Estrogen fluctuation and cessation of menstrual cycle during menopause linked to proatherogenic alterations in the lipid profile. Independent of the aging process, menopausal status is associated with increased serum levels of total cholesterol, LDL cholesterol, apolipoproteins and triglycerides, as well as reduced levels of high-density lipoprotein cholesterol (HDL-C). During menopause, particular attention should be given to women with conditions that are more closely linked to cardiovascular disease (CVD), such as dyslipidaemia, polycystic ovarian syndrome, premature menopause, early menopause, premature ovarian insufficiency and familial hypercholesterolemia [2]. In postmenopausal women, low-density lipoprotein cholesterol (LDL-C) levels increase by 10-15% due to a decline in estrogen-driven hepatic LDL receptor activity. Smaller, denser LDL particles exhibit an increased susceptibility to oxidative modification, making them more prone to retention within the arterial walls. This oxidative modification accelerates foam cell formation, contributing to endothelial dysfunction and arterial plaque development. As a result, these LDL subfractions play a crucial role in the progression of atherosclerosis and cardiovascular disease [3]. High-density lipoprotein cholesterol (HDL-C) declines by 5-10%, with a significant reduction in the cardioprotective HDL2 subfraction. Additionally, HDL's protective functions, such as its antioxidant capacity, become less effective [4, 5]. Increased VLDL secretion and decreased lipid clearance cause a 10-20% increase in triglyceride levels [6]. Postmenopausal women exhibit increased LDL/HDL and TC/HDL ratios, which serve as strong predictors of CAD-related mortality. For example, in Nepal, 18.4% of postmenopausal women had a TC/HDL ratio exceeding 5, indicating a high risk of cardiovascular disease [7]. In 2019, cardiovascular disease (CVD) was estimated to have caused approximately 17.9 million deaths, representing 32% of all global deaths. Over three-quarters of these occurred in low- and middle-income countries [8]. Dyslipidemia promotes endothelial dysfunction and thrombosis in concert with menopausal weight gain and adipokine dysregulation. Dyslipidaemia is the predictor of cardiovascular disease (CVD) and the major risk factor of coronary artery disease (CAD) the cause for increased morbidity and mortality among these women. In Nepal, women constitute more than half of the population, with a significant portion being over 50years. This age group tends to have lower physical activity levels and unhealthy dietary habits, increasing their risk of atherogenic effects. Since this is the typical age for menopause, there is growing concern that more Nepalese women may face complications related to cardiovascular disease [9]. Studies says that women with menopause have a 2-3 times greater risk of dyslipidaemia and required proactive, intensive monitoring. There have been limited studies from Nepal regarding the prevalence of pattern of dyslipidemia among post-menopausal women. Hence it is necessary to analyse the lipid abnormality in pre and post-menopausal women. Therefore, it is of interest to report the comparative study of dyslipidemia among pre and post - menopausal women.

## Materials and Methods:

The present hospital based cross sectional study was conducted on the patients who are attending the outpatient department of general medicine for their routine health check-up at Universal College of Medical Sciences and Teaching Hospital (UCMS & TH) Bhairahawa, Nepal were included in this study. The present study was approved by the institutional ethics committee, UCMS & TH. The experiments followed the amended Helsinki Declaration of 1975, revised in 2013. The Raosoft online sample size calculator was used to estimate the sample size. The minimum sample size of 197 was required for a population of 400 women who attended a general routine health check-up, considering a 95% confidence interval and a 5% margin of error [10]. However totally N=214 were recruited among them (n=107) were premenopausal and (n=107) were postmenopausal women. Postmenopausal women who have undergone hysterectomy, diabetes illness and who are taking lipid lowering drugs were excluded from this study.

## Sample collection:

After taking informed consent, 2ml of venous blood samples were collected in plain centrifuge tubes in the antecubital vein from all the participants after overnight fasting (8-12) hours. The blood was allowed to clot for 10 minutes and then centrifuged at 3000 rpm for 15 minutes at room temperature and analysed for lipid profile. Total Cholesterol (TC) was measured using established enzymatic methods of Allain *et al.* 1974 [11]. Triglyceride (TG) was assessed enzymatically [12] and High Density Lipoprotein Cholesterol (HDL-c) [13] was estimated by using a fully automatic Erba XL 200 analyzer. The Low Density Lipoprotein Cholesterol (LDL-c) and Very Low Density Lipoprotein Cholesterol (VLDL-c) were derived using Freidewald's formula [VLDL-c =TG/5; LDL-c = TC - (HDL + TG/5)] [14]. Dyslipidaemia was defined according to the third report of National Cholesterol Education Program Adult Treatment Panel (NCEP ATP III) guideline [15]. Based on the criteria hypercholesterolemia defined as Total cholesterol (TC) ≥ 200 mg/dl, Low Density Lipoprotein cholesterol (LDL-c) >100 mg/dl, Hypertriglyceridemia (TG) ≥150 mg/dl and High Density Lipoprotein cholesterol (HDL-c 40 mg/dl. The study groups were classified to without dyslipidaemia, isolated dyslipidaemia and mixed dyslipidaemia in pre and postmenopausal women. Isolated dyslipidaemia was defined as isolated hypercholesterolemia, isolated hypertriglyceridemia or isolated high LDL-c. Mixed dyslipidaemia was defined as the combination of two or more of high level of above mentioned serum lipid concentration. Without dyslipidaemia was defined the lipid profile was within the reference range of the said values. Atherogenic index of plasma (AIP) is a logarithmically transformed ratio of molar concentrations of triglycerides to HDL-cholesterol calculated as log10 (TG/HDL-C) [16]. Non-HDL-C was calculated by subtraction of HDL from the TC and ≥130 mg/dl was considered an elevated level of non-HDL-C [17].

## Statistical analysis:

The Statistical analysis was done by using SPSS software version 20. Categorical data were expressed as percentage. The variables were expressed as mean ± Standard Deviation (SD). Independent samples t-test (2 tailed) was used to compare means of variables and ANOVA used for Analysis of variance of the study groups. Two-sided P values < 0.05 were considered statistically significant. Pearson's correlation analysis was done to analyse the significant association between various parameters.

## Results:

The study was carried out in 214 healthy women, among them 107 were premenopausal and 107 were postmenopausal women. The average age of pre-menopause group of women was 35.34 ± 6.19 and in post-menopausal women was 57.87 ± 8.07. The pattern of serum dyslipidaemia among pre and post-menopausal women was identified. According to this pattern, without dyslipidaemia in premenopausal women was 36.4 % (n=39) which is absent in postmenopausal women. Among premenopause 26.1% (n=28) and in post menopause 35.5% (n=38) had isolated lipid profile abnormality, whereas mixed dyslipidaemia was 37.3% (n=40) among pre-menopause and 64.4% (n=69) among post menopause shown in Figure 1 (see PDF). Student t test showed that serum TC, LDL, VLDL and Non HDL levels were found to be significantly increased in postmenopausal women compared to premenopausal women (P value<0.001) showed in Table 1. The prevalence of isolated dyslipidaemia in premenopausal women was 26.10% (n=28) and mixed dyslipidaemia was 37.3 % (n=40). The prevalence of isolated dyslipidaemia in postmenopausal women was 35.5% (n=38) and mixed dyslipidaemia was 64.4 % (n=69) shown in Figure 1 (see PDF). In the present study, there was significant difference in the Age, Total cholesterol, LDL, HDL, VLDL and Non HDL between pre and postmenopausal women as shown in Table 1 (see PDF). As shown in Table 2 (see PDF) dyslipidaemia was found to be present in 81.77% (n=175) of the total study population. It is also noted that without dyslipidaemia absent in postmenopausal women. The graph shows that Non-HDL-C has a very strong correlation with total cholesterol (r = 0.975) and LDL-C (r = 0.930), both statistically significant (P < 0.0001) shown in Figure 2 (see PDF) and Figure 3 (see PDF). This means as non-HDL-C increases, both total cholesterol and LDL-C typically increase as well.

## Discussion:

Cardiovascular disease (CVD) stands as the foremost cause of death among women in the United States, largely attributable to the high prevalence of dyslipidemia [18]. About 80% of the 20.5 million fatalities in 2021 that were attributed to CVD recorded in low- and middle-income nations [19]. The prevalence of abnormal blood lipid profiles is increasing in developing nations due to their rapidly growing economies and changing lifestyles [20]. Our study shows prevalence of dyslipidaemia increased in postmenopausal women compared to premenopause. We also observed significant increase in TC, TG, LDL and VLDL in postmenopausal women compared to those in premenopausal. These findings agreed with similar studies of other regions worldwide [21]. These results were agreed with Berg *et al.* who also showed increased total cholesterol, Triglycerides and LDL-C in postmenopausal and menopausal transitional women compared to premenopausal women [22]. This may be due to the fact that reduced estrogen levels lead to an increase in total cholesterol and lipoproteins, resulting in a lipid profile that promotes atherogenic potential [23]. Clinical trials have demonstrated that medications aimed at lowering low-density lipoprotein cholesterol can decelerate the angiographic progression of coronary heart disease and reduce clinical events in both men and women [18]. In our study, without dyslipidaemia is none in post-menopausal women. And we also observed mixed dyslipidaemia was more prevalent in postmenopausal women when compared to premenopausal women. This also agrees with Bell *et al.* 2012 [24].

Studies show that HDL is decreased in post-menopausal women. However in our study the HDL is increased in postmenopausal women compared to pre menopause this also agreed by the study done by Fernandez *et al.* 2016, After menopause, visceral adipose tissue (VAT) increases markedly regardless of age, indicating that sex hormone levels play a key role in determining fat distribution in women. It is therefore possible that the low HDL-C concentrations observed by some researchers after menopause are not a direct result of menopause itself, but rather a consequence of increased VAT commonly seen in post-menopausal women. Conversely, in women whose VAT remains unchanged after menopause, HDL-C levels may remain stable or even increase, as demonstrated in the present study [25, 26]. When individual lipid levels fall within normal ranges, the LDL-c/HDL-c ratio has become a crucial biomarker for assessing cardiovascular risk. This ratio, which represents both atherogenic (LDL-C) and protective (HDL-C) cholesterol components, offers a dynamic evaluation of the balance of lipid metabolism [27, 28].

Our study revealed that although the LDL/HDL ratio increased in postmenopausal women, the difference was not statistically significant. Interestingly, when analyzing within the dyslipidemia pattern, the LDL/HDL ratio found to be increased in mixed dyslipidemia of premenopausal women compared to postmenopausal women. This could be attributed to lifestyle factors such as dietary habits, physical inactivity or hormonal fluctuations during the reproductive years that may influence lipid metabolism. Additionally, the use of hormonal contraceptives and stress-related metabolic changes might contribute to the altered lipid profile in premenopausal women. The atherogenic index of plasma (AIP) is increasingly recognized as a robust predictor of cardiovascular (CV) risk. This index integrates triglyceride (TG) and HDL-cholesterol (HDL-C) levels into a single metric, reflecting both lipid imbalance and lipoprotein particle size - a critical factor in atherosclerosis development. In fact, it has been proposed that low CV risk is associated with AIP values between - 0.3 and 0.1, medium CV risk with values between 0.1 and 0.24 and high CV risk with values above 0.24 [29]. When evaluating cardiovascular (CV) risk factors, the atherogenic index of plasma-a mathematical link between TG and HDL-C-has been effectively employed as an extra index [17]. Studies shows that AIP significantly increased in post-menopausal women however, in our study the AIP not significantly increased in pots menopausal women which is also agreed by Guo *et al.* 2020, this suggests the possibility of lifestyle or ethnic influences on the predictive usefulness of AIP [30]. Non-HDL cholesterol (non-HDL-C) encompasses all cholesterol contained within atherogenic lipoprotein particles, including LDL-C, lipoprotein (a), VLDL-C, VLDL remnants and intermediate-density lipoproteins (IDL-C). In recent years, increasing serum levels of non-HDL-C have attracted considerable attention as a more reliable predictor of cardiovascular risk compared to traditional lipid markers [31, 32- 33]. In our study non HDL significantly increased in postmenopausal women compared to premenopausal. Non HDL also significantly correlated with total cholesterol and LDL of postmenopausal women. This also agreed by Sigdel *et al.* 2012 [34]. This shows that postmenopausal hormonal alterations contribute to adverse lipid profile changes in women and may underlie the cause for elevated non-HDL cholesterol levels [35].

## Conclusion:

Dyslipidaemia is highly prevalent among post-menopausal women and serves as a significant risk factor for cardiovascular disease. Effective management of dyslipidaemia is essential for preventing both primary and secondary cardiovascular events, including myocardial infarction, ischemic stroke and coronary mortality. Implementing targeted interventions can help mitigate these risks and improve overall cardiovascular health in women.
